# O Que Prever da Vida Cardiovascular aos 85?

**DOI:** 10.36660/abc.20210138

**Published:** 2021-06-08

**Authors:** Wouter Kok

**Affiliations:** 1 Amsterdam University Medical Center Amsterdã Holanda Amsterdam University Medical Center , Amsterdã – Holanda

**Keywords:** Doenças Cardiovasculares, Fatores de Risco, Idoso, Serviços de Saúde para Idosos, Mortalidade, Peptídeo Natriurético-Tipo B

Embora haja muita literatura para a previsão de risco de doenças cardiovasculares e terapias preventivas, ^1^ as estimativas de risco são menos conhecidas para pacientes com idade >80 anos. É necessário suprir essa lacuna de conhecimento, pois o estado de saúde dos idosos com idade mais avançada terá cada vez mais impacto na assistência à saúde. ^2^

Um estudo detalhado realizado em Pequim por Zhu et al., ^3^ publicado nesta revista, descreve o risco em 5 anos de doença cardiovascular em 724 pacientes chineses muito idosos – todos com idade >80 anos, a maioria deles do sexo masculino. ^3^ Eles foram internados em um Departamento de Cardiologia Geriátrica, área que está em evolução a nível internacional. ^4^ Os motivos da internação estavam relacionados principalmente a doença arterial coronariana e controle da hipertensão; apenas alguns foram admitidos devido a doenças respiratórias ou do trato digestivo. Após um seguimento médio de 5,3 anos e taxa de seguimento de 98%, cerca de 50% dos pacientes morreram, a maioria deles devido a infecções, e apenas 1 em 16 pacientes de causa cardíaca. O estudo mostra que a morbidade cardiovascular e o risco de mortalidade por todas as causas nessa população podem ser previstos com sucesso pelos níveis do fragmento N-terminal do peptídeo natriurético tipo B (NT-ProBNP). ^3^

A predição de mortalidade por todas as causas e eventos cardiovasculares com baixos níveis de NT-proBNP tem sido realizada na população geral com idade de 50 a 89 anos, ^5^ e na população geriátrica com idade >80 anos. ^6 , 7^ Como deveríamos interpretar esses níveis baixos e o que eles predizem? Com base em outros estudos, parece que esses níveis mais baixos de NT-proBNP não apenas predizem a morte cardiovascular, mas também a não-cardiovascular. ^8^ Uma interpretação pode ser que as variações do NT-proBNP em níveis tão baixos são uma medida da idade biológica, refletindo as várias interações com o NT-proBNP. ^8^ Há outras medidas de vitalidade, como escores de fragilidade em pacientes idosos que também predizem eventos CV; portanto, esse conceito não é novo. ^9^ Além disso, na insuficiência cardíaca com frações de ejeção preservadas, estamos começando a ver que valores baixos de NT-proBNP mantêm seu valor preditivo para mortalidade por todas as causas, embora não haja certeza de que os desfechos serão cardiovasculares. ^10^ Por outro lado, a descoberta de que níveis muito mais elevados de NT-proBNP, como por exemplo na insuficiência cardíaca, nem sempre implicam em uma mortalidade por todas as causas muito alta ou imediata; isso é exemplificado em um estudo de pacientes idosos com idade ≥ 85 anos nos quais os níveis de NT-proBNP na faixa de 1707-9729 ng/L ainda estavam associados à sobrevida de 1 ano de quase 100%, enquanto somente os pacientes acima dessa faixa apresentaram aumento da mortalidade. ^11^ O risco final a ser previsto depende das distribuições de riscos adicionais. Portanto, o achado no artigo de Zhu et al., ^3^ de que níveis baixos de NT-proBNP predizem independentemente a mortalidade por todas as causas é o esperado, mas tem a contrapartida de que a maior parte da mortalidade foi não-cardiovascular, e o registro documentado de que quase todos os pacientes tinham fração de ejeção preservada no ecocardiograma. ^3^

Portanto, é um achado interessante que também os eventos cardiovasculares maiores (ECAM) não-fatais, que têm uma incidência muito maior do que a mortalidade cardiovascular, são previstos de forma precisa pelos baixos níveis de NT-proBNP. Na Tabela 3, os ECAM (n = 202) são apresentados com uma incidência de cerca de 1 em 4 pacientes (28%) após um seguimento médio de 5 anos; a maioria dos eventos envolvem síndromes coronárias agudas (incidência de 19%), e um pouco menos frequentes são os acidentes vasculares cerebrais (incidência de 5%). Sabe-se que essas incidências de ECAM aumentam exponencialmente com a idade, como visto em uma população britânica, onde pessoas com idade >80 anos têm um risco de incidência em 10 anos de 50% de doença cardiovascular – um composto de eventos coronários e cerebrais. ^12^ Devido à alta incidência de ECAM, a identificação de pacientes idosos com riscos intermediários desses eventos já teria implicações para a prevenção, e não somente para aqueles com maior risco de ECAM. Também pode ser interessante saber qual é a relação entre a morbidade cardiovascular (ECAM) e a mortalidade por todas as causas.

Uma interpretação tentadora dos níveis de NT-proBNP da Tabela 1 é que 4 categorias do NT-proBNP resumem os riscos de doença cardíaca coronária anterior, hipertensão, fibrilação atrial e diminuição da função renal, com o aumento de todos os riscos com os níveis mais altos de NT-proBNP. Os níveis de NT-proBNP não parecem refletir a presença de diabetes mellitus (distribuição igual) ou níveis de colesterol (que diminuem com elevação do NT-proBNP). Entretanto, um modelo de risco seria necessário para as estimativas de risco reais desses fatores.

Para aqueles interessados em terapias preventivas, os ECAM no estudo de Zhu et al. ocorreram apesar dos medicamentos protetores (70% dos pacientes receberam agentes antiplaquetários, 45% receberam estatinas, e 40% inibidores de ECA ou BRAs). Seria interessante avaliar as interações dos medicamentos após considerar toda a gama de risco de eventos CV (e não em relação aos tercis do NT-proBNP).

Pode-se concluir, com base no trabalho de Zhu et al., que a vida cardiovascular aos 85 pode ser prevista pelos baixos níveis de NT-proBNP, mas, ao mesmo tempo, devemos reconhecer que essa avaliação de risco não deveria depender somente dos pontos de corte do NT-proBNP.
