# Supplementary material for: O Que Prever da Vida Cardiovascular aos 85?
Source: Arq Bras Cardiol. 2021 Jun 8;116(6):1037–8. [Article in Portuguese] doi: 10.36660/abc.20210138 (PMC8288533; doi:10.36660/abc.20210138)
Supplement: Translation

# Translation: What to Expect from Cardiovascular Life at 85?

**Authors:** Wouter Kok

**Affiliations:** 1 Netherlands Amsterdam University Medical Center, Amsterdam - Netherlands

**Keywords:** Cardiovascular Diseases, Risk Factors, Health Services for the Aged, Mortality, Natriuretic Peptide, B-Type

While there is plenty of literature for risk prediction of cardiovascular disease and preventive therapies,^1^ risk estimates are less well known for patients aged > 80 years. There is a need to address this gap in knowledge, as the health status of very elderly people will increasingly impact on health care.^2^

A detailed study carried out in Beijing by Zhu et al., published in this journal, describes the 5-year risk of cardiovascular disease in 724 very elderly Chinese patients - all > 80 years old, most of them men.^3^ They were admitted to the geriatric cardiology department, something that is an evolving field at the international level.^4^ The reasons for hospital admission were mostly related to coronary artery disease and hypertension control; only a few were admitted for respiratory or digestive tract disease. After a median follow-up of 5.3 years and a follow-up rate of 98%, about 50% of the patients died, most of them from infections and only 1 in 16 patients from a cardiac cause. The study shows that cardiovascular morbidity and all-cause mortality risk in this population can be successfully predicted by the N-terminal pro-B-type natriuretic peptide levels (NT-proBNP).^3^

Predicting all-cause mortality and cardiovascular events with low levels of NT-proBNP has been done in the general population aged 50-89 years,^5^ and in the geriatric population >80 years.^6 , 7^ How should we interpret these low levels, and what do they predict? Based on other studies, it seems that lower levels of NT-proBNP not only predict cardiovascular but also non-cardiovascular death.^8^ One interpretation may be that variations in NT-proBNP at such low levels are a measure of biological age, reflecting the various interactions with NT-proBNP.^8^ There are other measures of vitality, such as frailty scores in elderly patients that also predict CV events, so this concept is not new.^9^ Also, in heart failure with preserved ejection fractions we are starting to see that low values of NT-proBNP maintain their predictive value for all-cause mortality, although there is no certainty that the outcomes will be cardiovascular ones.^10^ Contrarywise, the finding that much higher levels of NT-proBNP, for example in heart failure, do not always imply a very high or immediate all-cause mortality; this is exemplified in a study of elderly patients aged ≥85 years in whom a range of NT-proBNP levels of 1707- 9729 ng/L was still associated with a 1-year survival of almost 100%, while only patients with levels above this range showed increased mortality.^11^ The final risk to be predicted therefore probably depends on distributions of additional risks. Thus, the finding in the study by Zhu et al.,^3^ that low levels of NT-proBNP independently predict all-cause mortality is as expected, but it has the catch that most of the mortality was non-cardiovascular, and the documentation that shows that almost every patient had an echocardiogram with preserved ejection fraction.^3^

Therefore it is an interesting finding that also major adverse non-fatal cardiovascular events (MACEs), which have a much higher incidence than cardiovascular mortality, are well predicted by low NT-proBNP levels. In Table 3 the MACEs (n= 202) are shown with an incidence of about 1 in 4 patients (28%) after a median follow-up of 5 years; most events comprise acute coronary syndrome (19% incidence), and somewhat less frequent are cerebral stroke (5% incidence). These incidences of MACE are known to exponentially increase with old age, such as seen in a British population, where people aged > 80 years have a 10-year incidence risk of 50% of cardiovascular disease - a composite of coronary and cerebral events.^12^ Because of the high incidence of MACE, identifying elderly patients with intermediate risks of these events would already have implications for prevention, and not only for those with the highest risk of MACE. It also may be interesting to know what the relationship is between cardiovascular morbidity (MACE) and all-cause mortality.

A tempting interpretation of NT-proBNP from Table 1 is that 4 NT-proBNP categories summarize the risks of previous coronary heart disease, hypertension, atrial fibrillation, and decreased renal function, all increasing with higher NT-proBNP levels. The NT-proBNP levels do not appear to reflect diabetes mellitus (equal distribution) or cholesterol levels (decreasing with higher NT-proBNP). However, a risk model would be needed for the actual risk estimates of these factors.

For those interested in preventive therapies, the MACEs in the study by Zhu et al. occurred despite protective medications (70% of patients received antiplatelet agents, 45% statins, 40% ACE inhibitors or ARBs). It would be interesting to assess medication interactions after considering the full range of the risk of CV events (not only in relation to NT-proBNP tertiles).

It can be inferred from the work of Zhu et al. that cardiovascular life at 85 may be predicted by low levels of NT-proBNP, but at the same time, we should acknowledge that this risk assessment should not depend on the cutoff levels of NT-proBNP alone.
